# The Bath of Decreasing Temperature in the Treatment of Typhoid Fever

**Published:** 1878-01

**Authors:** M. H. Gartin

**Affiliations:** Dover, Illinois


### THE BATH OF DECREASING TEMPERATURE IN THE TREATMENT OF TYPHOID FEVER.

By M. H. Garten, M. D., Dover, Illinois.

I do not make any claim to original ideas in the treatment of typhoid fever, as set forth in this article, but merely wish to relate the history of a case which, to me, appears to be of unusual interest, and whose active symptoms were mitigated under the influence of the bath of decreasing temperature.

Mrs. E. C——, aged 20, in her first pregnancy, nursed her husband during an attack of typhoid fever. I was called to see her Sept. 2d, 1877. She stated that she had not been well for several days, that her flesh was sore, and that at times she was chilly and feverish.

I found her with a pulse of thirty beats, tongue slightly coated, pain in the head and back, slight abdominal tenderness, and the bowels loose. Having conducted her husband through his fever, I naturally suspected that this case was one of the same character, and gave tincture of gelseminum, sub-nitrate of bismuth and opium.

Sept. 4th.—The pulse was 120, the bowels moving less frequently, free epistaxis at times, and an annoying cough. No appetite. The bismuth and opium were continued, but instead of gelseminum, I gave dilute muriatic acid and tincture of digitalis.

Sept. 6th.—No marked change.

Sept. 7th.—Morning temperature in axilla, 103 3-5; pulse, 120. Evening temperature, 105; pulse, 118. Bowels loose. Free epistaxis during the day and cough; tongue dry and fissured; milk or beef tea at regular intervals unless the patient sleeps. The nurse is instructed to omit the medicine rather than the nourishment.

Sept. 8th.—Morning temperature, 102 4-5; pulse, 114; evening temperature, 105; pulse, 120. The patient to be sponged freely with tepid water, morning and evening.

Sept. 9th.—Morning temperature, 103; pulse, 118; evening temperature, 104: pulse, 120. Slightly delirious, bowels loose, increased abdominal tenderness, tongue dry and sore, teeth heavily coated with sordes. A teaspoonful of alcohol in a wine-glassful of sweetened water every two hours is added to the treatment.

Sept. 10th.—Morning temperature, 103; pulse, 120; evening temperature, 106; pulse, 118. Rested poorly last night; more active; frequent change of position, or picking at the bedding; when interrogated, she answers correctly, yes or no; cough quite annoying, dry and harsh; voids feces and urine in bed; protrudes tongue with difficulty.

Sept. 11th.—Morning temperature, 104; pulse, 130, and tremulous; rested but little last night; bowels moving freely; much prostration.

Thus, from day to day, I observed my patient gradually losing her hold on life, until she was but the shadow of her former self. So far as I was able to form an opinion, the medication availed but little, and yet the exalted temperature, so rapidly doing the work of destruction, had to be reduced. The use of quinine, as an antipyretic in other cases, had disappointed me, and hence I determined to make trial of the bath, though as I had never before made use of it in the treatment of disease, it was not without some foreboding that the trial was now made. Ziemssen's method of treating typhoid fever was received by me as I fear country physicians receive too many good suggestions, as a probably efficient method in the hands of its author, or in the practice of a large hospital, but wholly inadequate to the purposes of a busy practitioner. At that time I never thought it possible for me to make it available or dreamed of ever daring to employ it.

Having procured a rubber bath-tub, it was placed at the bedside, and partly filled with water at a temperature of 94°, 10° less than that of the patient. She was placed in the tub with a sheet, folded two or three times, arranged so as to prevent the cold water from coming in direct contact with her person. The tub was covered with a sheet, so that she was directly incased, with the exception of the head. Upon the outer sheet the water, fresh from the well, was poured, and left to find its way into the tub. This was continued until she complained of being chilly, when she was removed to the bed and lightly covered, having remained in the bath twenty-five minutes. The temperature of the water was reduced from 94° to 70°.

I should mention the fact that at all times I was governed in the regulation of the temperature by the patient's sensation of cold, instead of by the instruction to reduce the temperature to 68°. Some of the baths given in this case were not reduced to a temperature below 78°, yet the wished-for results were obtained, viz., a decided reduction of temperature, and a complaint on the part of the patient that she was cold. After the bath, the patient's temperature registered 102 2-5°, instead of 104°; and she rested comfortably in bed, without becoming exhausted, as I had feared.

At evening I found she had improved during the day; pulse, 112; temperature, 103—a decrease of 3° since last evening. Another bath was therefore given.

Sept. 12th.—Morning temperature, 101 3-5; pulse, 114; evening temperature, 102 2-5; pulse, 120. Two baths given to-day. She is quite rational; tongue moist and cleaning; cough not so frequent; asks to use the bed-pan; does not object to taking nourishment.

Sept. 13th.—Morning temperature, 102 2-5; pulse, 112; evening temperature, 103 1-5; pulse, 120. Is doing well. Two baths given in the day.

Sept. 14th.—Morning temperature, 100 3-5; pulse, 114. Is quite bright, and improving her time in removing the sordes from her teeth.

Dr. E. F. Ingals, Assistant Editor of this journal, saw Mrs. C—— with me, last evening and this morning; also concurring in my diagnosis. As her temperature was so much lower than on the previous morning, no bath was given. At evening, however, I found the temperature to be 103 3-5, a little more than on the previous evening; pulse, 120. Another bath was given to-night.

Sept. 15th.—Morning temperature, 102 3-5; pulse, 114; the temperature was higher than on the previous morning. Evening temperature, 102 2-5; pulse, 106. Two baths given to-day. The patient is improving.

Sept. 16.—Morning temperature, 99; pulse, 96; evening temperature, 102; pulse, 106. She has a desire for nourishment, and prefers beef tea.

Sept. 21st.—From the 16th to the 21st inst., the temperature ranged between 99° morning, and 102 3-5° evening.

Sept. 22d.—Morning temperature, 98½; pulse, 92; gave bath at 3 p. m. to-day. The temperature just before the bath was 100 3-5; just after, 99°. The temperature of the water (92°) was reduced to 75°; 7 p. m., temperature, 100; pulse, 96.

Since this date, the bath has been given before the hour for the rise of temperature, and at no time has the temperature advanced above 99 3-5.

Sept. 26th.—Temperature, 98½; pulse, 82. Every symptom is propitious, and a rapid convalescence anticipated.

Sept. 27.—I was summoned in haste at 2 a. m., and found labor had supervened. It slowly advanced until 8 a. m., when the fœtal head having descended, I delivered her with forceps, of a living, skeleton-like child, which appeared to be about eight months old. The mother passed through her labor far better than was expected, losing but very little light-colored blood.

Sept. 28th.—She rested well during the night, and requested me to state that she is convalescent, and is indebted for it to the bath.

Oct. 10th.—Rapid convalescence from both the febrile and the puerperal states.

After this experience, I feel convinced that merely placing a patient in water is not so much to be dreaded as, on first impression, one is induced to believe. While this mode of treatment requires more time than the visit and prescription of routine practice, it is safe, efficient, and pleasant for the patient, wholly under the control of the physician, and easily comprehended by an intelligent nurse. Quinine, on the other hand, given in large doses in typhoid fever, is unreliable, dangerous, and capable of doing irreparable injury. The same may be said of all arterial sedatives. I am of the opinion that the treatment here recommended would be equally valuable in typho-pneumonia. In Mrs. C.'s case, the dry, hacking cough ceased to be annoying after I had resorted to bath treatment.

The details given above of the treatment of this case, are taken from notes carefully made by myself at the time of each visit, and I shall not regret their publication if they shall, in any degree, contribute toward relieving others of that timidity with which many are disposed to regard new methods of treatment—those especially which have come to us from abroad. I might add, that the case is published also with a view to inducing others to make a fair trial in typhoid fever of the bath of decreasing temperature.

